# A new species of *Linopherus* (Annelida, Amphinomidae) from Beibu Gulf, South China Sea

**DOI:** 10.3897/zookeys.640.9619

**Published:** 2016-12-13

**Authors:** Yue Sun, Xinzheng Li

**Affiliations:** 1 Institute of Oceanology, Chinese Academy of Sciences, 7 Nanhai Road, Qingdao 266071, China; 2 University of Chinese Academy of Sciences, Beijing 100049, China; 3 Laboratory for Marine Biology and Biotechnology, Qingdao National Laboratory for Marine Science and Technology, Qingdao, Shandong, 266070, China

**Keywords:** Fireworm, new species, polychaete, Pseudeurythoe, systematics, taxonomy

## Abstract

*Linopherus beibuwanensis* **sp. n.** is described based on six specimens deposited in the Marine Biological Museum of the Chinese Academy of Sciences, Qingdao, collected from the Beibu Gulf (Tokin Gulf), South China Sea. The new species differs from all other *Linopherus* species by the shape of prostomium and caruncle. The posterior margin of prostomium is bilobed; the caruncle arises medially and confluently from the posterior margin of the prostomium, joining together with prostomium. A key to distinguish the known species of the genus is provided.

## Introduction

Polychaetes belonging to the family Amphinomidae are commonly known as fireworms due to the burning sensation caused by the calcareous harpoon notochaetae (Fauchald, 1977). The amphinomids are globally distributed and common in shallow tropical and subtropical waters (Kudenov 1995): *Linopherus abyssalis* (Fauchald, 1972a), *Linopherus hemuli* (Fauchald, 1972b), and *Chloeia kudenovi* Barroso & Paiva, 2011 are examples of recently reported species from abyssal depths and polar areas (Barroso 2011; Fauchald 1972a, b).

Species of the genus *Linopherus* Quatrefages, 1865 have been referred as members of *Pseudeurythoe* Fauvel, 1932. Fauchald (1972a) described a new species from abyssal depths and reviewed the species of *Pseudeurythoe*. In 1977, he treated *Pseudeurythoe* as a synonym of *Linopherus*. Since then *Linopherus* has been accepted by subsequent authors and in the present work (San Martín 1986; Salazar-Vallejo 1987). *Linopherus* differs remarkably from the other genera of the family in having species without caruncle or with a reduced caruncle, and branchiae that are limited to the anterior part of the body. The morphology of the prostomium, the development of prostomial appendages, caruncle, parapodia, and the number of branchiae are important in species identification (Kudenov 1995). To date, six species of *Linopherus* have been reported from the coastal waters of China (Sun, Lei and Zhou 2008): *Linopherus ambigua* (Monro, 1933), *Linopherus paucibranchiata* Fauvel, 1932, *Linopherus hirsuta* (Wesenderg-Lund, 1949), *Linopherus oligobranchia* (Wu, Shen & Chen, 1975), *Linopherus microcephala* (Fauvel, 1932), and *Linopherus spiralis* (Wesenderg-Lund, 1949).

When the authors examined the Amphinomidae archived in the Marine Biological Museum of Chinese Academy of Sciences (MBMCAS), Institute of Oceanology, Chinese Academy of Sciences (IOCAS), six specimens of *Linopherus* were separated out. Careful examination revealed that these specimens represented a new species. The new species is described here, and a key to worldwide species of *Linopherus* is provided.

## Material and methods

The specimens were collected during the late 1950s to early 1960s from Beibu Gulf (Tonkin Gulf), northern South China Sea. All specimens are deposited in the **MBMCAS**, preserved in 75% ethanol solution. Microscopy observations and drawings were made using a Zeiss Stemi 2000-C stereomicroscope equipped with an AxioCam MRc 5 digital camera.

## Systematics

### Family Amphinomidae Lamarck, 1818 Genus *Linopherus* Quatrefages, 1865

#### Linopherus beibuwanensis sp. n.

Taxon classificationAnimaliaAmphinomidaAmphinomidae

http://zoobank.org/83C424FA-951B-4F12-8CEC-B6CD6088AD20

Fig. 1

##### Material examined.

Holotype, MBM010010, Beibu Gulf, 21°15'N, 108°06'E, 91m, sandy mud, coll. Xiutong Ma, 11 Feb 1959. Paratypes, MBM200142, Beibu Gulf, 18°30'N, 107°00'E, 66m, sandy beach, coll. Zhengang Fan, 10 Sep 1960; MBM200143, Beibu Gulf, 18°00'N, 107°45'E, 90m, silty mud, coll. Ruiping Sun, 9 Apr1962; MBM200144, Beibu Gulf, 18°00'N, 108°00'E, 93m, sandy mud, coll. Xiutong Ma, 11 Dec 1959; MBM200145, Beibu Gulf, 18°30'N, 107°00'E, 66m, sandy beach, coll. Xiutong Ma, 9 Dec 1962; MBM200122, Beibu Gulf, 18°00'N, 107°00'E, sandy mud, coll. Zhengang Fan, 14 Feb1960.

##### Diagnosis.

Prostomium globular, posterior margin bilobed, with two pairs of eyes, anterior pair semicircular in shape. Medial caruncle conspicuous, arising from and confluent with posterior prostomial margin, medial lobe projecting above paired lateral lobes. Parapodia biramous, rami widely separated, with single dorsal and single ventral cirrus. Branchiae dendritic, present from chaetiger 3 onwards, with more than 40 pairs.

##### Description.

Holotype (MBM010010) incomplete, lacking posterior part, 17 mm long, 2 mm wide excluding chaeta, with 45 chaetigers. Body elongate, nearly rectangular in cross section, tapering posteriorly. Color in alcohol pale yellow, without color pattern.

**Figure 1.**
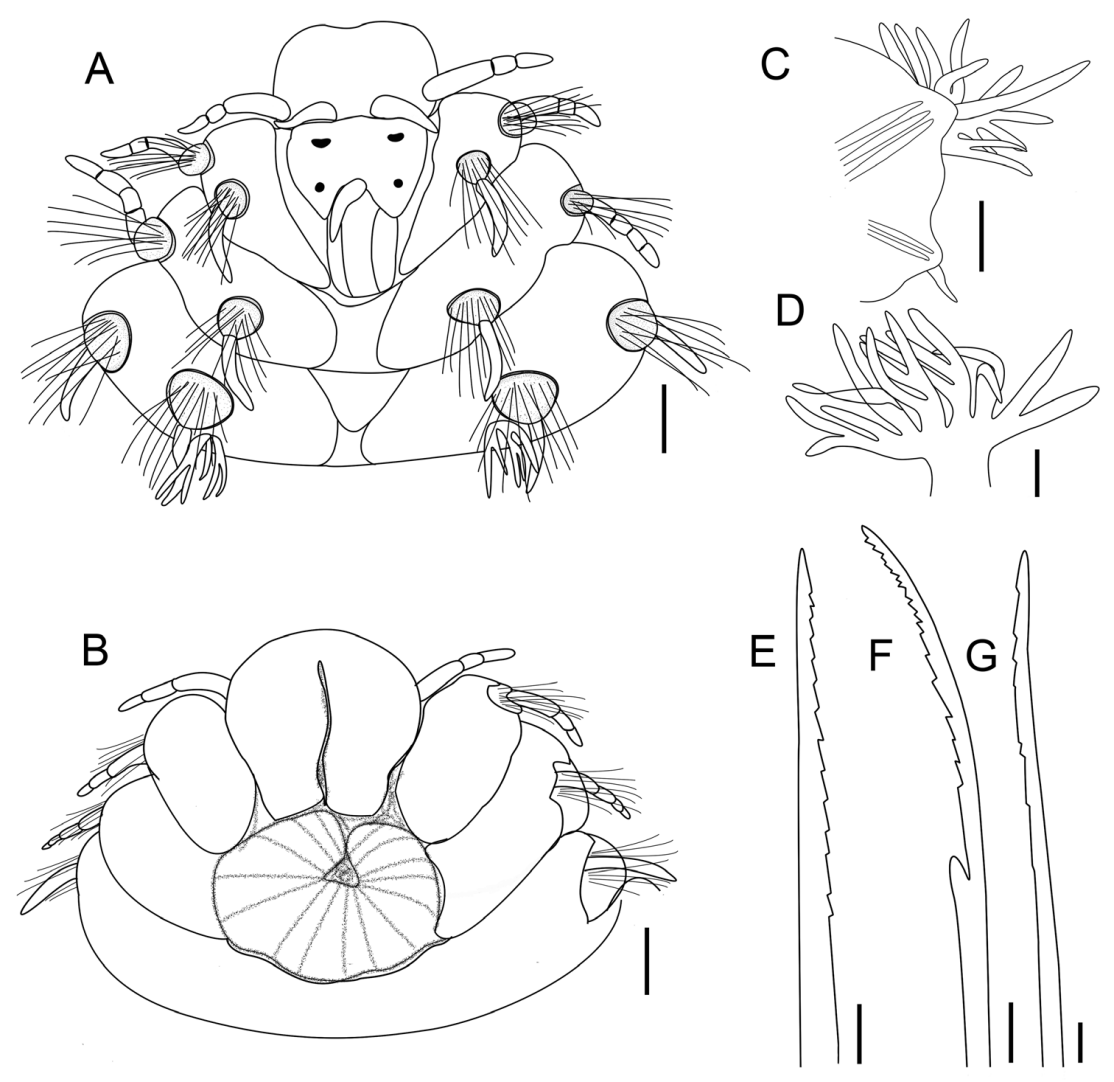
*Linopherus beibuwanensis* sp. n. **A** Anterior end in dorsal view **B** Anterior end in vental view **C** left parapodia of chaetiger 14 in frontal view **D** branchiae of chaetiger 28 **E** harpoon notochaeta **F** forked neurochaeta **G** slender capillary chaeta. Scale bars: **A–B**, 200 μm; **C–D**, 250 μm; **E–F**, 50 μm; **G**, 20 μm.

Prostomium (Fig. 1A) divided into two parts by transverse groove. Anterior lobe rounded, with pair of cirriform antennae dorsally and similar pair of palps laterally, palps with three distinct articulations. Posterior lobe heart-shaped, bilobed along posterior margin, with median antenna and two pairs of reddish eyes. Anterior pair of eye spots semicircular in shape and large, posterior pair of eye spots rounded and small. Median antenna conical, slightly shorter than paired antennae, located at posterior margin of prostomium. Buccal opening occupying two chaetigers (Fig. 1B).

Caruncle rectangular and conspicuous, medial lobe projecting above paired lateral lobes. Caruncle restrict to chaetiger 1, equal in length to posterior prostomial lobe of prostomium, reaching to anterior margin of chaetiger 2, arising medially and confluently with posterior prostomium (Fig. 1A).

All parapodia biramous, with chaetiger 1 greatly reduced, chaetiger 2 forming first dorsally complete ring. Parapodia with slender cirri (Fig. 1A, C), both notopodia and neuropodia well-developed, chaetal sac forming low rounded lobe. First two parapodia with longer and more conspicuous cirri than other parapodia; ventral cirri of chaetiger 2 longest, each with four articulations (Fig. 1A). Succeeding parapodia with tapering and rather short ventral cirri.

Branchiae present from chaetiger 3, located on posterior face of each notopodia (Fig. 1A), arising as tufts, dendritically branched, decreasing along body and disappearing at approximately chaetiger 42; chaetiger 1 branchiae with five terminal filaments, chaetiger 17 branchiae with 29 or 26 terminal filaments, chaetiger 28 branchiae with 15 filaments (Fig. 1D), chaetiger 42 branchiae with 6 or 7 filaments.

Notochaetae of three types: stout harpoon chaetae, numbering 13–24 per fascicle (Fig. 1E); slender capillary chaetae (Fig. 1G), faintly serrated, without basal spur, many broken; notoacicula, numbering 3–4 per fascicle, hastate (Fig. 1C). Neurochaetae of three kinds: forked chaetae, short limb reduced as spur, with thin shafts and long, distally serrated cutting margins (Fig. 1F); slender, capillary chaetae, smooth; neuroacicula, numbering 2–3 per fascicle, slightly hastate, slender than notoacicula (Fig. 1C).

##### Variations.

All specimens examined are incomplete lacking the posterior part. Specimen size varies from 1.9 to 2.5 mm in maximum width. Branchial chaetiger range varies from 3 to 31–40. The maximum number of branchial filaments varies from 20–29. One female paratype (MBM200143) with eggs in posterior coelomic cavity, ranging in diameter from 71μm to 90 μm.

##### Etymology.

The species is named after Beibuwan, the Chinese name for the Beibu Gulf (Tonkin Gulf), South China Sea.

##### Distribution.

Only known from Beibu Gulf, South China Sea.

##### Remarks.

*Linopherus beibuwanensis* sp. n. is referred to *Linopherus* because of the arrangement of branchiae and the reduced but characteristic caruncle. *Linopherus beibuwanensis* sp. n. is unique in this genus for the combined shape of its prostomium and the caruncle. While the posterior prostomial margin is straight (not bilobed) in most *Linopherus* species, their caruncles are also either absent or reduced. In the latter case, species with reduced caruncles typically exhibit a transverse groove that appears to isolate the caruncle from the prostomium, which is usually partly or completely concealed by the second chaetiger (see Langerhans 1881; Fauvel 1932; Monro 1933, 1937; Treadwell 1941; Wesenberg-Lund 1949; Knox 1960; Fauchald 1972a, b; Kudenov 1975; Wu et al. 1975; Kudenov and Blake 1985; San Martín 1986; Salazar-Vallejo 1987).

*Linopherus beibuwanensis* sp. n. is similar to *Linopherus abyssalis* in having the posterior prostomial margin bilobed. The new species can be distinguished from the latter by the presence of eyes and arrangement of branchiae; the former has two pairs of eyes and more than 40 pairs of branchiae, while the latter has no externally detectable eyes and only 5-6 pairs of branchiae.

Caruncle morphology appears to be an essential character which can be used to distinguish the species of *Linopherus*. However, *Linopherus* species are small in size, and so their caruncle morphology is imperfectly known since it is usually concealed by constriction of the anterior chaetigers. Clearly, further research on this taxon is necessary.

### Key to species of *Linopherus* (modified from Fauchald 1972 and Salazar-Vallejo 1987).

**Table:** 

1	Branchiae present from chaetiger 2 continuing to end of body	***Linopherus reducta* (Kudenov & Blake, 1985)**
–	Branchiae present from chaetiger 3 or 4	**2**
2	Branchiae present from chaetiger 3	**3**
–	Branchiae present from chaetiger 4	**15**
3	Eyes absent	**4**
–	Eyes present, one or two pairs	**5**
4	Several pairs of branchiae until end of body	***Linopherus tripunctata* (Kudenov, 1975)**
–	Only six pairs of branchiae	***Linopherus oligobranchia* (Wu, Shen & Chen, 1975)**
5	First pair of eyes semicircular	**6**
–	First pair of eyes rounded	**8**
6	Posterior margin of prostomium bilobed, more than 40 pairs of branchiae	***Linopherus beibuwanensis* sp. n.**
–	Posterior margin of prostomium not bilobed, less than 40 pairs of branchiae	**7**
7	Six pairs of branchiae, caruncle absent	***Linopherus fauchaldi* San Martín, 1986**
–	About 23 pairs of branchiae, caruncle present, small and rounded	***Linopherus microcephala* (Fauvel, 1932)**
8	Anterior lobe of prostomium conical, not expanded anteriorly	***Linopherus kristiani* Salazar-Vallejo, 1987**
–	Anterior lobe of prostomium rounded, anteriorly expanded	**9**
9	One pair eyes	**10**
–	Two pairs of eyes	**11**
10	Caruncle present, cirri of second chaetiger longer than others	***Linopherus paucibranchia* (Fauvel, 1932)**
–	Caruncle absent, cirri of second chaetiger as long as others	***Linopherus hirsuta* (Wesenberg-Lund, 1949)**
11	More than 20 pairs of branchiae present	**12**
–	Maximally 16 pairs of branchiae present	**14**
12	Branchiae present only on anterior chaetigers, eyes indistinct	***Linopherus ambigua* (Monro, 1933)**
–	Branchiae present in all but first two and last few chaetigers, eyes distinct	**13**
13	Caruncle absent, eyes inconspicuous	***Linopherus spiralis* (Wesenberg-Lund, 1949)**
–	Caruncle present, eyes conspicuous	***Linopherus oculata* (Treadwell, 1941)**
14	13-16 pairs of branchiae present, caruncle present	***Linopherus annulata* (Hartmann-Schröder, 1965)**
–	Maximally seven pairs of branchiae present, caruncle absent	***Linopherus canariensis* Langerhans, 1881**
15	More than 40 pairs of branchiae present, eyes distinct, caruncle absent	***Linopherus acarunculata* (Monro, 1937)**
–	Less than 10 pairs of branchiae, eyes absent, caruncle present	**16**
16	Seven pairs of branchiae present, subdistally inflated (hastate) acicular chaetae absent	***Linopherus minuta* (Knox, 1960)**
–	Less than 7 pairs of branchiae present, subdistally inflated (hastate) acicular chaetae present	**17**
17	Five or six pairs of branchiae present, dorsal cirri absent in branchial chaetigers, caruncle in deep pocket at posterior margin of prostomium, small and rounded	***Linopherus abyssalis* (Fauchald, 1972)**
–	Five pairs of branchiae present, dorsal cirri absent in branchial chaetigers, caruncle button-shaped	***Linopherus hemuli* (Fauchald, 1972b)**

## Supplementary Material

XML Treatment for Linopherus beibuwanensis
